# Meanings and Roles of Great-Grandparenthood: A Scoping Review

**DOI:** 10.1093/geront/gnae181

**Published:** 2024-12-16

**Authors:** Zuzana Talašová, Adéla Souralová

**Affiliations:** Department of Sociology, Faculty of Social Studies, Masaryk University, Brno, Czech Republic; Department of Sociology, Faculty of Social Studies, Masaryk University, Brno, Czech Republic

**Keywords:** Great-grandparenthood, relationship between great-grandparents and great-grandchildren, role in family

## Abstract

**Background and Objectives:**

Great-grandparenthood brings a new internal dynamic to intergenerational relationships in which contact between 4 generations is now the norm. In this scoping review, we sought to identify the roles of great-grandparents and what those roles entail.

**Research Design and Methods:**

We performed a review using PRISMA-ScR to identify peer-reviewed studies investigating the roles of great-grandparents. The reviewed articles were selected from 4 databases. The article selection conditions were met by 23 studies that used quantitative or qualitative methods. The studies had to be published in English; the selected publications spanned 8 countries.

**Results:**

The scoping review covers a collection qualitative and quantitative research with different types of respondents (great-grandchild, grandparents, great-grandparents, etc.). An important element was a statement on great-grandparenting from at least 1 of the 4 generations. We selected 23 articles from an initial selection of 176 studies. The studies were heterogeneous in conceptual frameworks, collection methods, and interview conduct, with qualitative methods predominating over quantitative methods.

**Discussion and Implications:**

Based on research on great-grandparenting, the roles of great-grandparents can be categorized as continuing, insignificant, or significant. Each role category has a specific function and each represents a different meaning for the great-grandparents within the family. Several factors and constraints frame the great-grandparent roles. Based on the research results, some characteristics are shared by all role categories despite the dividing elements.

A higher life expectancy increases “the co-survivorship across generations” (Bengtson, 2001, p. 6), and “long-lasting intergenerational relationships across three and even four generations are now a frequent and relevant phenomenon” (Matos & Neves, 2012, p. 204). Demographic change has critically altered the family system, structure, and dynamics (Barranti, 1985; Hagestad, 2008; Keck & Saraceno, 2008). The family structure resembles “a beanpole” (Bengtson et al., 1996) where horizontal ties among siblings, cousins, aunts, and uncles decrease whereas vertical ties increase. Harper (2006, p. 181) described this phenomenon as the “verticalization of the family structure,” meaning that people spend “more time as parents and children, more time as grandchildren, and more time as great-grandchildren/great-grandparents” (see also Knipscheer, 1988). In the same vein, Soliz and Lin (2014) discuss how “the family is shifting from intragenerational to intergenerational,” highlighting that intergenerational relationships are becoming increasingly important, often at the expense of relationships within the same generation. Furthermore, this shift underscores the reality that individuals now have more opportunities to experience a variety of family roles throughout their life course, as relationships between different generations take on a more central role in family life. Among these roles are those formed across generations, such as the roles of grandparents, great-grandparents, grandchildren, and great-grandchildren.

The social sciences have responded to these profound family changes with extensive research on grandparenting, with articles on grandparenthood and grandparent–grandchild relations regularly appearing in journals such as Gerontologist (e.g., Barrett & Gunderson, 2021; Kennedy, 1990; Somary & Strieker, 1998). In 1985, Hagestad (p. 36) noted that in contemporary society, “grandparents range in age from 30 to 110, and grandchildren range from newborns to retirees,” highlighting the significant diversity within grandparenthood. Given this broad age span, it is unsurprising that research on grandparenthood has traditionally focused on classifying styles and roles (Neugarten & Weinstein, 1964; Robertson, 1976; Cherlin & Furstenberg, 1986; Kornhaber, 1996; Mueller et al., 2002), examining influences (Matos & Neves, 2012; Tinsley & Parke, 1987; Tomlin, 1998), and exploring the meanings and dimensions of grandparenthood (Kivnick, 1982, 1983; Robertson, 1976).

Recent research on grandparenthood has expanded significantly, introducing new perspectives and issues (Harper & Levin, 2005; Szinovacz, 1998; Hayslip & Fruhauf, 2019; Attias-Arber & Timonen, 2012; Attias-Donfut & Segalen, 2002; Timonen, 2020). This expansion reflects the individualized society and the theoretical framework of individualization developed by Beck (1992), Beck & Beck-Gernsheim (2001), and Giddens (1991). As Kemp (2004, p. 501) notes, the evolving focus on self-fulfillment and institutionalized individualism heightens interest in how grandparents and grandchildren negotiate their roles and relationships. Consequently, the experience of grandparenting, including its negotiations and meanings, can no longer be confined to a few predefined styles or roles (Arber & Timonen, 2012; Timonen, 2020). Instead, grandparenting should be viewed as shaped by both sociodemographic and economic influences as well as by grandparents’ own agency (Timonen, 2020, p. 2). Reflecting advances in family studies, current research emphasizes the performative and relational aspects of grandparenting, focusing on voluntary family ties and the dynamic nature of triadic relationships among grandparents, adult children, and grandchildren (Arber & Timonen, 2012).

In parallel, though less intensively, research on great-grandparenthood has also begun to slowly develop during the same period. The study of great-grandparenthood and the relationships between great-grandparents and great-grandchildren has been largely overshadowed by the extensive focus on grandparenthood. Although existing research on grandparenthood provides a useful foundation for exploring great-grandparenthood, it does not fully capture the unique perspectives of great-grandparents in their new role, despite its close relation to that of grandparents. It is crucial for researchers to devote more attention to great-grandparenthood as a distinct family role, one that carries its own set of expectations, meanings, and practices. How does the role of great-grandparents differ from that of grandparents when it comes to intergenerational caregiving? What are the expectations placed on great-grandparents within the dynamics of multigenerational families? Although this study does not aim to answer these questions directly, it seeks to stimulate future research into these and other inquiries regarding great-grandparenthood. By offering a foundational overview of the current research, this study encourages further exploration beyond the dominant focus on grandparenthood and leverages valuable insights from the extensive scholarship on grandparenthood. These insights can offer important perspectives on this emerging field, particularly in terms of the diversity of great-grandparenting, as well as the agency, performativity, and relationality inherent in this role.

The aim of this scoping review is to investigate the current research on great-grandparenting and to identify the characteristics and meanings of great-grandparent roles. We asked the following questions: *What is the role of great-grandparenting in current social science research according to English-language publications? What are the characteristics of these roles? How are these roles interpreted?* We performed a review using PRISMA-ScR to identify peer-reviewed studies investigating the role of great-grandparents. As a result of our analysis of the 23 selected publications, we identified three primary roles that emerged directly from our examination: the continuing grandparenthood role, the insignificant role, and the significant role. We elucidate the characteristics of these roles and the factors that influence them.

## Method

### Research Design

A scoping review provides a quick overview of key concepts and the main resources available. It presents a comprehensive overview of the nature and characteristics of research and makes it possible to identify areas where further research is needed (Arksey & O’Malley, 2005). The PRISMA-ScR (Preferred Reporting Items for Systematic Reviews and Extension of Meta-Analyses for Scoping Assessment) checklist is used here to enable he review. PRISMA-ScR provides a clear scope covering rationale, goals, criteria, resources, search strategies, etc. (Tricco et al., 2018). This approach is known for its flexibility and ability to quickly map the available literature in new or underexplored areas. Although it may be considered limited in the scope of included studies, we took several steps to expand our search strategy. We used multiple databases, included a broad range of relevant keywords, and repeatedly tested their combinations to ensure the highest possible relevance of the included articles. Through this process, we achieved a representative overview that accurately reflects the state of research on great-grandparenthood and provides a solid foundation for future, more extensive research in this area.

The primary responsibility for designing the search strategy was held by the lead author, who consulted with the second author regarding the selection of keywords, databases, and combinations of Boolean operators. Four major academic databases were selected—Scopus, Web of Science, Google Scholar, and EBSCO Discovery Services—which were considered representative to ensure broad coverage of relevant literature in the study of great-grandparents’ roles.

### Search Strategy

As part of the search strategy, four academic databases were searched: *Scopus*, *Web of Science*, *Google Scholar*, and *EBSCO Discovery Services*. When searching, the Boolean operators “AND” and “OR” were added for keywords and subject headings related to great-grandparents, such as: “great-grandparents,” “great-grandparenthood,” “great-grandchildren,” “great-grandmother,” “great-grandfather,” “four-generation family,” and “multigenerational family.” The highest results were found for the keyword “great-grandparents,” with authors using the term directly in the title or abstract of the paper. Further searches using the other key terms added little to the already identified literature, and these particular search results were not used in the scoping review as they were not written in English. The Boolean operator “NOT” was mostly used to exclude literature that found research on “grandparenting.” If the literature on grandparenting had not been excluded, the literature review results would have been many times higher and would not have been useful for this scoping review on the role of great-grandparenting. The search was conducted from September 18 to December 10, 2023, when the literature search was verified using keywords.

### Search Criteria

Peer-reviewed publications were included in the search. The search methods were search repetition and confirmation. The year of publication was not essential, given the low number of publications on the topic. The study criteria included articles dealing with grandparenting issues, where the grandparent’s perspective was not the sole focus. At least one generation commented on great-grandparenting. All texts were in English. The studies had to be supported by quantitative or qualitative research to determine the role of great-grandparents. Publications were excluded if they: (a) did not refer to great-grandparents, (b) did not mention the role of the great-grandparent, (c) were not in English, or (d) did not include a reflection on great-grandparenting. In conducting the scoping review, 23 studies were selected from an initial pool of 176 articles that met all the inclusion criteria. The process of excluding articles was carried out in several steps: (a) The first step involved the removal of duplicate records, resulting in the exclusion of 24 articles. These articles were identified as duplicates, appearing in multiple databases or through repeated searches. (b) The second step was assessing the relevance of the articles based on their titles and abstracts. In this phase, 105 articles were excluded because they did not align with the study’s focus. These articles, for example, did not directly address the role of great-grandparents, were focused on other aspects of family relationships, or covered broader topics without a specific emphasis on great-grandparents. (c) In the third step, 47 articles were fully reviewed to determine their suitability based on the complete text. Out of these, 24 articles were excluded because they did not meet all the predefined criteria. The reasons for exclusion included: not referring to great-grandparents, not discussing the role of great-grandparents, not being published in English, and lacking reflection on the topic of great-grandparenthood.

Research was not divided on the basis of whether it was quantitative or qualitative, primarily because there were not many publications on great-grandparenting. The primary aim was to find as much published research as possible dealing with the role of great-grandparenthood or addressing this role even marginally.

### Extracting Data for Charting

Data were extracted from qualitative and quantitative studies by one author (Z. T.). The author examined the given data in several stages over a 4-month period. The data were independently reviewed by the second author (A. S.). During the identification of the studies by the first author (Z. T.), there was a discussion with the second author (A. S.) concerning agreement on the criteria and the selection of publications that ensured value in the review process.

The following items were identified in studies: (a) lead author, (b) year of publication, (c) data collection methods, (d) participant characteristics (sample size, age, gender), (e) identification of the role of the great-grandparent (continuing grandparenthood role, significant role, or insignificant role), and (f) study location (only country was listed, due to the lack of information in some studies). These data were organized into tables (Table 3) and analyzed to identify patterns and common elements across different studies. The mapping process allowed us to contextualize the roles of great-grandparents in various cultural and social contexts, thereby contributing to a better understanding of the dynamics and significance of great-grandparenthood in modern families. During this process, we also identified and recorded factors that influence the role of great-grandparents, such as health status, geographical distance, economic status, and gender. These findings were key to the synthesis of data and the creation of a comprehensive picture of the various roles of great-grandparents as described in the available literature. Thus, data mapping allowed for the creation of a structured and systematic overview of the roles of great-grandparents and their impact on intergenerational relationships, which is essential for understanding these dynamics within the broader context of societal changes.

### The Phase of Interpreting Results

We created tables summarizing the characteristics of quantitative (Table 1) and qualitative studies (Table 2), which provide a quick overview of the main features of each study (Table 3). Based on the extracted data, we identified key themes related to the roles of great-grandparents (Table 4). These themes include the level of involvement of great-grandparents, their perception of their role, and their influence on family dynamics. By employing thematic analysis, we identified patterns and trends across the studies, which involved comparing different definitions and descriptions of the roles of great-grandparents, examining factors affecting their involvement (e.g., geographical distance), and analyzing interactions between great-grandparents and other generations.

**Table 1.** Characteristics of the Reviewed Quantitative Studies

Author (year)	Data collection methods	Sample size, age, gender	Great-grandparenthood role	Study location
**Castañeda-García et al. (2021)**	Questionnaire	*N* = 68; age 70–89; 76.5% women	Continuing grandparenthood	Spain
**Castañeda-García et al. (2017)**	Questionnaire	*N* = 46; age 70–98; not specified, more women than men	Insignificant	Spain
**Drew and Silverstein (2004)**	Questionnaire	*N* = 188; age 56–96; 64.4% women	Continuing grandparenthood	United States
**Evans et al. (2021)**	PSID^1^^)^	Not specified	Continuing grandparenthood	United States
**Even-Zohar and (Tzurit) Garby (2016)**	Questionnaire	*N* = 103; age 66–94; 76.7% women	Continuing grandparenthood/significant	Israel
**Knigge (2016)**	GENLIAS^2^^)^	Not specified; 100% men	Continuing grandparenthood	Netherlands
**Roberto and Skoglund (1996)**	Questionnaire	*N* = 52; age 18–21; 83% women	Insignificant/continuing grandparenthood	United States
**Xu et al (2022)**	CHARLS ^3^^)^	*N* = 3,389; age (⌀) 57–58; 49.2% women	Continuing grandparenthood	China

^1)^PSID = panel study of income dynamics.

^2)^GENLIAS = digitized information from Dutch marriage certificates.

^3)^CHARLS = China health and retirement longitudinal study.

**Table 2.** Characteristics of the Qualitative Studies Reviewed

Author (year)	Data collection methods	Sample size; age; gender	Great-grandparenthood role	Study location
**Albrecht (1954)**	Interviews, observations, and possibly surveys or questionnaires (mix methods)	*N* = not specified; 65+; not specified	Significant	United States
**Barer (2001)**	Interview technique + open-ended, structured questioning	*N* = 122; 85+; 77% women	Insignificant	United States
**Bean et al. (2014)**	Interviews	Not specified	Significant	United States
**Brannen (2006)**	Biographical-interpretive narrative interview	*N* = 71 family members; age and gender not specified	Continuing grandparenthood/significant	United Kingdom
**Burton (1992)**	Field observation, focus groups, life-history interviews, participant-observation in family activities, and in-depth interviews	*N* = 60; 43–82; 83% women	Continuing grandparenthood	United States
**Doka and Mertz (1988)**	Interview	*N* = 40; 71–90; 88% women	Significant	United States
**Dolbin-MacNab et al. (2009)**	Interviews	*N* = 20*; 19–52; 50% women	Significant	United States
**Lamas-Abraira (2021)**	Interview + participant observation	Not specified	Significant	Spain, Poland, China
**Miron et al. (2019)**	Interviews	*N* = 16*; 18–38; 50% women	Significant	United States
**Schuler and Dias (2020)**	Interview + textual analysis	*N* = 10; 74–97; 40% women, 10% men + great-grandchildren	Continuing grandparenthood	Brazil
**Schuler (2022)**	The use of surveys and research studies	Not specified	Significant	Brazil
**Schuler and Brito Dias (2023)**	Interviews	*N* = 22; 60–97; 14% women, 9% men + family members)	Significant	Brazil
**Reese and Murray (1996)**	Interviews	*N* = 16; 75–87; 100% women	Significant	United States
**Powell (2018)**	Interviews	*N* = 17; age not specified; 88% women	Significant	United Kingdom
**Wentowski (1985)**	Interviews	*N* = 19; 66–92; 100% women	Continuing grandparenthood	United States

^*^Participants who were raised by grandparent and great-grandparent.

**Table 3.** Description of the Roles of Great-Grandparenthood Based on a Publication

Great-grandparenthood role	Author (year)	Definition
Insignificant	**Castañeda-García et al. (2017)**	The role of great-grandparents is insignificant because their interactions with great-grandchildren are less frequent and more formal, influenced by factors like age, health issues, and geographical distance.
	**Roberto and Skoglund (1996)**	The role of great-grandparents is insignificant due to limited and infrequent interactions with great-grandchildren, resulting in minimal influence on their lives. Geographic distance and health limitations often restrict their role in family life.
	**Barer (2001)**	The role of great-grandparents is often insignificant due to the physical and emotional distance that develops between generations and their limited ability to actively engage in the lives of their great-grandchildren. As they age and face health limitations, their role becomes more symbolic, with the primary responsibility shifting to the intervening generation of parents.
The common feature of the definitions that describe the role of great-grandparents as *insignificant* is the emphasis on limited and infrequent interactions with great-grandchildren. These limitations are often caused by factors such as age, health issues, and geographical distance, leading to minimal influence on their great-grandchildren’s lives. As a result, their role becomes more symbolic rather than active, with primary responsibility shifting to intervening generations, such as parents.		
Continuing grandparenthood	**Castañeda-García et al. (2021)**	The role of great-grandparents continues the care and support they provided as grandparents. Although their interactions with great-grandchildren are less frequent and less demanding, they still contribute to family gatherings and maintain the continuity of family values and traditions.
	**Drew and Silverstein (2004)**	The role of great-grandparents continues the role of grandparents because it contributes to a unified family identity that supports psychological well-being and maintains family traditions and relationships across generations.
	**Evans et al. (2021)**	The role of great-grandparents continues the transmission of education and socioeconomic advantages across generations, even though their influence is mediated by intervening generations like parents and grandparents.
	**Even-Zohar and (Tzurit) Garby (2016)**	The role of great-grandparents continues the role of grandparents by maintaining emotional support and relationships across generations, contributing to a sense of belonging and family continuity.
	**Knigge (2016)**	The role of great-grandparents continues through the transfer of durable resources, such as property and family reputation, influencing the status level of great-grandchildren across generations, even without direct contact.
	**Roberto and Skoglund (1996)**	The role of great-grandparents continues the role of grandparenting by contributing to the transmission of family values and support, even with limited contact, thereby maintaining the continuity of family relationships across generations.
	**Xu et al (2022)**	The role of great-grandparents is a continuation of the grandparent role, particularly in the context of intergenerational caregiving. They extend their responsibilities by caring for both their elderly parents and grandchildren, reflecting ongoing family support and the cultural value of filial piety. This evolution highlights their enduring significance in the family’s caregiving structure, maintaining their influence across generations.
	**Brannen (2006)**	The role of great-grandparents continues the care and support they provided to their children and grandchildren, ensuring the continuity of family relationships. Through this transfer of material and caregiving resources, they maintain family culture and identity across generations.
	**Burton (1992)**	The role of great-grandparents continues the care and support they provided as grandparents, especially when they take on the responsibility for grandchildren whose parents are unable to fulfill their duties. This ensures stability and continuity for the next generation.
	**Schuler and Dias (2020)**	The role of great-grandparents continues the role of grandparenting by passing down family values, traditions, and life experiences, thereby maintaining the continuity of family relationships across generations.
	**Wentowski (1985)**	The role of great-grandparents continues the role of grandparenting as they model their behavior based on their experiences as grandparents, and they continue to provide emotional support and maintain family bonds, even though their ability to fulfill this role may be limited by age and distance.
The common feature of the definitions that describe the role of great-grandparents as *a continuation* of grandparenting is the emphasis on preserving family values, traditions, and support across generations. Although interactions with great-grandchildren may be less frequent and intense, great-grandparents still contribute to a unified family identity and ensure the continuity of relationships. The transfer of material resources, education, and life experiences further strengthens their role in maintaining family stability and cohesion.		
Significant	**Even-Zohar and (Tzurit) Garby (2016)**	The role of great-grandparents is important because it provides meaning and fulfillment, enhancing their quality of life through positive emotions like joy and pride from interactions with their great-grandchildren.
	**Albrecht (1954)**	Great-grandparents provide important emotional support, contribute to the transmission of family values and traditions, and by staying involved in the lives of younger generations, they maintain a sense of usefulness and connection.
	**Bean et al. (2014)**	The role of great-grandparents is significant because they provide stability and care for children who have experienced neglect or trauma, helping them overcome negative experiences. Their care brings a sense of purpose and fulfillment, positively impacting both the children and the great-grandparents themselves.
	**Brannen (2006)**	The role of great-grandparents is important because it contributes to the intergenerational transfer of values, material resources, and care, strengthening family relationships and ensuring the continuity of family culture and identity across generations.
	**Doka and Mertz (1988)**	The role of great-grandparents is important because it provides emotional fulfillment and a sense of personal and family renewal, bringing a feeling of continuity and significance to their lives.
	**Dolbin-MacNab et al. (2009)**	The role of great-grandparents is important because they provide children with stability and security, offering them a sense of unconditional love and a strong emotional bond that is crucial for their healthy development and emotional well-being.
	**Lamas-Abraira (2021)**	The role of great-grandparents is important because it contributes to family stability and cohesion through the transfer of care and the maintenance of family bonds and traditions across generations.
	**Miron et al. (2019)**	The role of great-grandparents is important because they contribute to maintaining family cohesion and provide emotional support, helping young adults cope with the challenges of dementia within the family.
	**Schuler (2022)**	The role of great-grandparents is important because they provide emotional support, maintain family traditions and values, and contribute to the care of younger generations, thereby strengthening family cohesion.
	**Schuler and Brito Dias (2023)**	The role of great-grandparents is important because they pass on values, traditions, and family heritage to their descendants, ensuring the connection and continuity of family identity across generations.
	**Reese and Murray (1996)**	The role of great-grandparents is important because it allows individuals to find meaning and transcendence in life by passing on their values, wisdom, and family stories to their great-grandchildren, thereby ensuring the continuity of family traditions.
	**Powell (2018)**	The role of great-grandparents is important because they provide physical and emotional support in multigenerational families, contributing to family stability and cohesion. They also help bridge generational gaps and promote the exchange of values across generations.
The common feature of definitions that highlight the role of great-grandparents as *important* is the emphasis on their ability to provide emotional support, maintain family values and traditions, and contribute to the stability and cohesion of the family. Great-grandparents ensure the continuity of family identity across generations, and their care brings meaning and fulfillment not only to themselves but also to the younger generations. This role is also significant for the transmission of values, love, and security, which is crucial for the healthy development and well-being of all family members.		

**Table 4.** Great-Grandparent and Great-Grandchild Relationship

Great-grandparenthood	Continuing	Insignificant	Significant
**Tends to maintain a relationship with each generation**	1	1	2
**Assumes responsibility for great-grandchildren if a parent or grandparent is unable to care (illness, work, etc.)**	2	1	2
**Has a sense of responsibility**	2	1	3
**Emphasizes identical religious form (if individuals are religious)**	2	2	2
**Pride and emotional attachment to all generations prevails**	1	1	1
**Remote social connection only**	3	3	1
**Sharing your own value system**	1	1	3
**Women are expected to help in the home during illness or crisis; Men are expected to help financially when needed**	2	2	3
**Meeting on special occasions (birthdays, holidays, Christmas, etc.)**	1	1	1
**Interaction with great-grandchildren**	1	1	3

The results of this analysis were presented in the form of a narrative summary, which provides a comprehensive view of the roles of great-grandparents as described in the current literature. To better illustrate these results, we used graphical elements that depict the frequency of various roles of great-grandparents across the studies. Through this approach, we ensured that the analysis phase of our scoping study not only thoroughly reflects the methodological framework proposed by Arksey & O’Malley (2005) but also provides a coherent overview of current research and identifies key areas for further exploration.

### Limitations of the Search Strategy

Although the search strategy was designed to maximize the inclusion of relevant studies, we restricted ourselves to peer-reviewed publications in English, which may impose certain limitations on the scope of the review. The search was also limited to four databases, which may have resulted in the omission of some relevant studies published in other databases or languages. Nevertheless, we believe that the chosen approach provided a sufficiently representative overview of the available literature, aligning with the objectives of this study.

## Result

**Figure 1.**
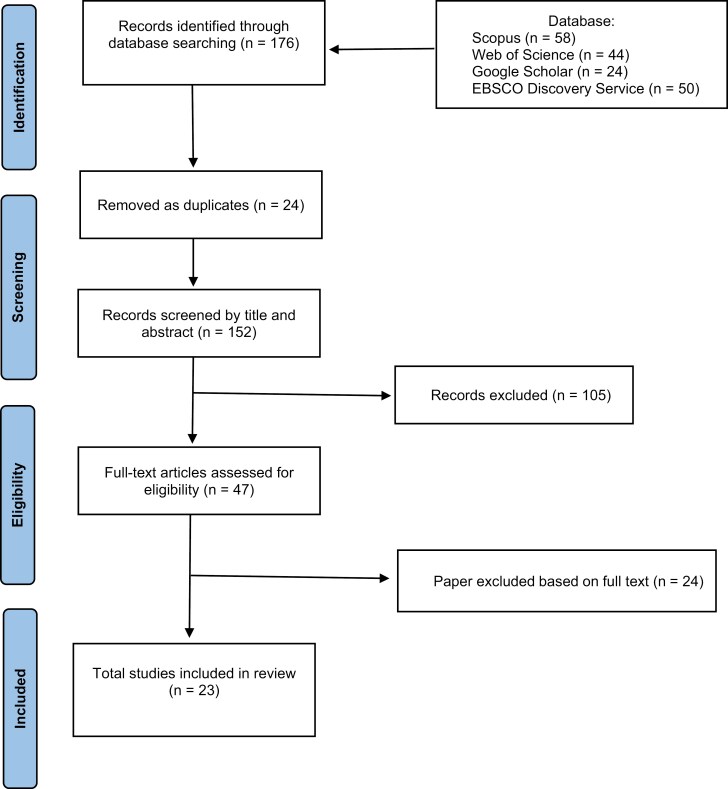
PRISMA diagram flow chart of the inclusion process.

The literature selection based on the chosen criteria is shown in Figure 1. A total of *n* = 176 publications dealing with (or mentioning) the topic of great-grandparenthood were searched. First, *n* = 24 (13.6%) publications were excluded due to duplication. Next, *n* = 105 (59.7%) publications were excluded on the basis of title and abstract indicating they were not relevant to the study. The total number of full-text articles assessed for eligibility was *n* = 47 (26.7%). Based on the criteria, *n* = 24 (13.6%) more studies were excluded. Finally, n = 23 (13.1%) full-text publications met the criteria and were selected for the scoping review. Of those, *n* = 8 (34.8%) studies used quantitative methodology and *n* = 15 (65.*2%)* studies used qualitative methodology.

In particular, the 2016 research by Ahuva Even-Zohar and Ayala (Tzurit) Garby should be noted. Their publications dealt mainly with the roles of great-grandparents with a specific regional focus on Israel. Their texts were instrumental in understanding and identifying the roles of great-grandparents.

### Characteristics of Evidence

**Table 1** shows the characteristics of the *n* = 8 (34.8%) publications with quantitative methodology. Most of the quantitative studies collected data using a questionnaire survey *n* = 5 (62.5%). The remaining *n* = 3 (38.5%) quantitative studies used longitudinal research: Evans et al. (2021) used the panel study of income dynamics; Knigge (2016) used digitized information from Dutch marriage certificates; Xu et al. (2022) used the China Health and Retirement Longitudinal Study. Sample size characteristics and age and gender were not reported for two of these studies. The studies were conducted in the US *n* = 3 (37.5%); Spain *n* = 2 (25%); Israel *n* = 1 (12.5%); the Netherlands *n* = 1 (12.5%); and China *n* = 1 (12.5%). In terms of sample size, the range was very diverse. In terms of indicating a given characteristic, there was a range of 46–3,389 respondents. Most respondents were women. Explicit statements by respondents regarding the role of the great-grandparent represent: insignificant role—*n* = 2 (25%); continuing grandparenthood—*n* = 7 (87.5%); significant role—*n* = 2 (25%). Although two roles are listed for some studies, they are considered individually. This means that the total number for a given role is not equal to the number of studies (e.g., the quantitative number of studies is 8, but the number of roles is 11).

Table 2 shows the characteristics of the *n* = 15 (65.2%) publications with qualitative methodology. Most of the studies collected data using an interview *n* = 14 (*93%)*; additional research methods were added to some interviews. Schuler’s (2022) research, *n* = 1 (7%), used surveys focusing on exploring the phenomenon of population aging in Brazil and research studies focusing on grandparenthood in Brazil and the US. Sample size and characteristics (age and gender) were not reported for three of these studies. The studies were conducted in the US *n* = 9 (60%); UK *n* = 2 (13%); Brazil *n* = 3 (20%); Spain *n* = 1 (7%); Poland *n* = 1 (7%); and China *n* = 1 (7%). As with the quantitative study, the sample of respondents is quite diverse, working with a range of 10–122 interviewees. Overall, women outnumbered men as respondents (when respondents were specified). Explicit statements by respondents regarding the role of the great-grandparent reflected an insignificant role—*n* = 1 (7%), continuing grandparenthood—*n* = 4 (27%), or a significant role—*n* = 11 (73%). As with the quantitative studies, the number of roles listed is greater than the number of studies. The study interviews collected opinions from different generations. A large part of the reported data notes comparisons of great-grandparents with grandparents.

### Exploring the Role

The studies listed in Table 1 draw upon the questionnaires, which typically had two parts. The first part included questions on age, gender, health status, number of children, grandchildren and great-grandchildren, and geographical distance. The second part varied by study. Castañeda-García et al. (2017) and Castañeda-García et al. (2021) focused on the interaction between great-grandparents and great-grandchildren; Even-Zohar and (Tzurit) Garby (2016) focused on assessing individuals’ subjective perceptions of quality of life. Roberto and Skoglund (1996) took a different approach in their research by having young adult great-grandchildren, complete questionnaires and assess their role in their relationship with their great-grandparents. Drew and Silverstein (2004) presented a different questionnaire, exploring the psychological well-being of great-grandparents. Three scales of inquiry were identified: self-esteem, depression, and affective balance. Respondents were searched through universities (Castañeda-García et al., 2017; Roberto & Skoglund, 1996) and organizations (Drew & Silverstein, 2004; Even-Zohar & (Tzurit) Garby, 2016); one study did not provide this information (Castañeda-García et al., 2021).

Table 2 shows how the qualitative research data was collected. Respondents were recruited through snowballing techniques (Barer, 2001; Lamas-Abraira, 2021), social networks (Brannen, 2006; Lamas-Abraira, 2021), organizations such as social services and communities (Brannen, 2006; Burton, 1992; Doka & Mertz, 1988; Dolbin-MacNab et al., 2009; Miron et al., 2019; Wentowski, 1985), and by phone (Schuler & Brito Dias, 2023); some studies did not provide recruitment information (Albrecht, 1954; Bean et al., 2014; Powell, 2018; Schuler, 2022).

As we observed, data were collected primarily through interviews with the research representing the diverse focus of a given interview. The studies are based on one or a combination of the following techniques: semi-structured interview techniques (Barer, 2001; Bean et al., 2014; Doka & Mertz, 1988; Dolbin-MacNab et al., 2009; Lamas-Abraira, 2021; Schuler & Dias, 2020), biographical-interpretive narrative interview (Brannen, 2006), field observation (Burton, 1992), focus groups (Burton, 1992; Miron et al, 2019), participant observation (Burton, 1992; Lamas-Abraira, 2021), in-depth interview (Burton, 1992), and analysis of the textual and graphic material from children’s books about great-grandparents (Schuler & Dias, 2020). The interviews were recorded using notes and then transcribed when further coded (coding methods were not always specified). Further coding sought common themes and patterns when these themes were independently identified by co-researchers.

An important element in the research was the individual’s retrospective. In-depth interviews conducted with great-grandparents provided insight into their family background and life experiences. The identification of the role was made explicit in the text. The authors identified one or more roles of great-grandparenting (e.g., one respondent might state that the role was a continuation of grandparenting and another might state that it was a completely new role in their life).

### Gender Predominance in Research

In the quntitative and qualitative research reported in Tables 1 and 2, we identified a predominance of female respondents. It should be noted that in the listed reviews, there were studies focused on male respondents (Knigge, 2016) or on female respondents (Reese & Murray, 1996; Wentowski, 1985), and studies where respondents were not specified (Albrecht, 1954; Bean et al., 2014; Brannen, 2006; Evans et al., 2021; Lamas-Abraira, 2021; Schuler, 2022). Only two studies had equal numbers of male and female respondents (Dolbin-MacNab et al., 2009; Miron et al., 2019). The study was aimed at understanding the role of grandparents in relation to their great-grandchildren and the distribution of participants by gender was not a deliberate choice.

## Discussion

There are several possible reasons that women predominated in the studies: (a) the influence of demographics; (b) matrilineal advantages; (c) life expectancy; and (d) women were more accessible to research than men.

1) *Influence of demographics = *may be due to the demographic composition of the study population, reflecting a higher number of women who meet the inclusion criteria.
2) *Matrilineal benefits = *studies show that great-grandmothers are more likely to have closer relationships with their great-granddaughters than great-grandchildren. This is consistent with the theory of matrilineal advantage. Matrilineal advantage refers to the observed pattern where the quality of the bond between grandchildren and great-grandchildren is stronger and closer on the mother’s side than on the father’s side (Chan & Elder, 2000).
3) *Life expectancy = *feminization of aging phenomenon, as most of the study participants were married or widowed. Women usually have a longer life expectancy than men (OECD, 2019; WHO, 2024). It should be added that there are regional and global differences in life expectancy and this information can change over time depending on social, economic, and health conditions.
4) *Approach to research = *Women traditionally play a more active role in childcare and family relationships in many cultures. Therefore, the influence of traditional gender roles and social norms and expectations may be prevalent. Women are often seen as caregivers and nurturers, which may have led to a greater interest in participating in studies related to family and intergenerational relationships (Fetterolf & Rudman, 2014; Hochschild & Machung, 1989; Souralová, 2019).

In this section, we focus on analyzing the factors that influence the role of great-grandparents in families. We find that these factors are very similar to those that affect the role of grandparents; however, some key differences persist. Notably, there is an absence of social perception and normative views on great-grandparenthood, which contrasts with the more clearly defined and homogeneous concepts of grandparenthood.

### Factors Affecting the Role of a Great-grandparent

The factors that influence the relationship between great-grandparents and great-grandchildren and the fulfillment of great-grandparenthood are very similar to the factors that influence grandparenthood. One major factor that is lacking in great-grandparents is the social perception (normative view) of great-grandparenthood. Social ideas about grandparenthood can be found, especially the homogeneity of fulfilling “grandparenthood” (Hasmanová Marhánková & Štípková, 2018; Noriega et al., 2017; Souralová, 2019; Vidovićová et al., 2015). Current widespread societal perceptions (“devoted grandmothers”) are at odds with reality (see Petrová Kafková, 2010). The factors that influence multigenerational interaction include:

#### Health status

The great-grandparents’ advanced age and chronic health conditions may interfere with their mobility and their ability to spend time with family members (e.g., holidays with great-grandchildren, family events). According to Doka and Mertz (1988) and Wentowski (1985), the higher the age and the poorer the health, the less frequent the social activities with great-grandchildren (Drew & Silverstein, 2004). Doka and Mertz (1988) reported slight differences in the age of great-grandparents. In their study, great-grandparents who were “younger in age” (approximately 78 years old) reported closer relationships with their great-grandchildren than great-grandparents who were “older in age” (approximately 90 years old). Interactions with great-grandchildren are more “sedentary” (communication via phone, communication via storytelling, etc.) yet they can help to bridge the generation gap and the connection between the two generations (Schuler & Dias, 2020). If there is no independent communication with great-grandchildren, an emotional distance from the youngest generation persists (Wentowski, 1985). An important family ritual that creates communication with great-grandchildren and mutual solidarity is Sunday lunch (Schuler & Brito Dias, 2023).

#### Geographical distance

The distance between the residence of the great-grandparents and the great-grandchildren is an important factor that determines their interaction with each other. Evidence suggests that the smaller the distance between homes, the more frequent the interaction between great-grandparents and great-grandchildren (e.g., picking up great-grandchildren from school; see Drew & Silverstein, 2004; Evans et al., 2021). This suggests that proximity plays a role in facilitating intergenerational activities. Although proximity may facilitate contact, it is not the sole determinant of contact between great-grandparents and great-grandchildren (Even-Zohar & (Tzurit) Garby, 2016).

#### Economic status

Socioeconomic factors play a significant role in the family practices of great-grandparents. For example, for grandparents, the lack of affordable childcare means that parents often rely on grandparents to care for their children (e.g., Arber & Timonen, 2012; Hasmanová Marhánková & Štípková, 2018). The importance of economic status is also reflected in great-grandparents with greater financial resources, who are more able to financially support grandchildren and great-grandchildren (Doka & Mertz, 1988; Roberto & Skoglund, 1996).

#### Gender

The influence of gender is reflected in the area of which great-grandchildren the great-grandparents meet and where family practices take place. Gender represents a key role in grandparenting with regard to the care of great-grandchildren (Hasmanová Marhánková, 2015; Hasmanová Marhánková & Štípková, 2018; Souralová, 2019; Souralová & Žáková, 2020). The few available sources on grandparenting suggest that gender is already a minor influence with respect to the distribution of caregiving roles. A focus on the role of grandmothers, with regard to cultural assumptions, shows that they are the ones who care for the grandchildren (continuing maternal roles), whereas grandfathers are the ones who pass on wisdom and traditions (Hasmanová Marhánková & Štípková, 2018; Možný, 1999). In the position of great-grandmother, some respondents report experiencing “grandfathering,” focused on communication and passing on values and traditions to great-grandchildren. Thus, the caregiving role seems to be blurring. In terms of grandparenting, the literature is very limited and so far suggests a continuing grandparenting role (Schuler & Dias, 2020; Wentowski, 1985, etc.).

#### Family traditions and rituals

Engaging in family traditions and rituals can strengthen the bonds between great-grandparents and great-grandchildren and help build strong relationships. These rituals and traditions can influence the role of the great-grandparent, whatever that role may be. Even if it is an unimportant role, great-grandparents may be “forced” into contact with the great-grandchild (such as at Christmas and birthday parties; Even-Zohar & (Tzurit) Garby, 2016; Roberto & Skoglund, 1996). These family rituals and traditions are often associated with emotions and memories and can represent a stable and continuous element in the lives of individuals. Although personal identity may be suppressed by this, family identity is reinforced (Bernardes, 1985; Calhoun et al., 2005).

#### Bridge between generations

The quality of the relationship between a great-grandparent and a great-grandchild is dependent on mediation between them (with the help of grandparents and grandchildren; Drew & Silverstein, 2004; [Bibr CIT0018][Bibr CIT0048]). This mediation is also influenced by the type of lineage (e.g., gender). Parents are the main mediators, though grandparents can be equally important. Cross-generational alliances may occur, with the youngest member serving as the negotiator (see Souralová & Žáková, 2020). For the time being, it cannot be stated that the same negotiation patterns are found in every four-generation family. As with grandparents, parental divorce represents a major intervention in the relationship between great-grandparents and great-grandchildren, especially on the father’s side (Hasmanová Marhánková & Štípková, 2018; Timonen et al., 2009). However, although some great-grandparents reported a negative relationship with their adult child, they were able to have positive relationships with their grandchildren and were thus able to establish a relationship with their great-grandchildren (Even-Zohar & (Tzurit) Garby, 2016). The hypothesis of matrilineal benefits has been supported by most of the available literature on great-grandparenting (e.g., Doka & Mertz, 1988; Schuler & Dias, 2020b; Wentowski, 1985).

In this section, we also summarize and synthesize the main roles of great-grandparents identified in the review, categorized into three groups: the continuing role of a grandparent, an insignificant role, and a significant role. Each of these roles has its specific functions and distinct importance for great-grandparents within the family.

### Continuing Grandparenthood

In continuing grandparenthood, the role of great-grandparents is still important, but only as an extension of the role of grandparents, not as a new function. Overall, the role of the great-grandparent was seen as a continuation of the grandparent role, but with some limitations and changes due to age and circumstances (Burton, 1992; Roberto & Skoglund, 1996). Respondents reported treating their great-grandchildren as grandchildren and modeled their behavior on their earlier role as the grandparent (Wentowski, 1985). The activities of great-grandparents may be similar to those performed by grandparents, but they are often less intense. For example, great-grandparents may practice traditional crafts, tell family stories, or share historical experiences with great-grandchildren. In this way, they pass on heritage to their children and introduce them to family history (Drew & Silverstein, 2004; Evans et al., 2021; Schuler & Dias, 2020).

Great-grandparents reported feeling isolated from the younger generation and feeling dependent on the “middle generation” to be connected to their great-grandchildren (Drew & Silverstein, 2004; Schuler & Dias, 2020; Wentowski, 1985). The authors suggested that this variable influences aspects of investment in the role of parents, grandparents, and great-grandparents and has an impact on the psychological well-being of the individual. Studies also report that the greater the geographic distance from the great-grandchildren, the lower the role salience (Brannen, 2006; [Bibr CIT0018][Bibr CIT0050]). The role of the great-grandparent is considered less significant than the role of the grandparent, even if the generations see each other daily, as there is little verbal interaction or joint activities between the great-grandparents and great-grandchildren (Castañeda-García et al., 2021). However, great-grandparents play a key role in how great-grandchildren learn about aging and can be important to the well-being of the family as support persons for grandparents and parents (Knigge, 2016).

### Insignificant Role

With the “insignificant” great-grandparent role, the role of the great-grandparent is marginal. Overall, it must be acknowledged that it is questionable whether the role is considered insignificant. Rather, there are new findings regarding the role of the great-grandparent in the family. It should be noted that the role of the great-grandparent appears to be ambiguous and poorly defined. Research by Barer (2001), Castañeda-García et al. (2017), and Roberto & Skoglund (1996) indicated that the great-grandparent–great-grandchild relationship is distant, characterized by limited contact. This places the role of great-grandparents closer to formal or distant roles in the family. Overall, studies show that great-grandparents had a rather marginal role in the family, especially in comparison to the role of grandparents. Respondents often reported limited social engagement with their great-grandchildren, mainly due to geographical distance, with contact being more ritualistic, such as during holidays and birthdays (Castañeda-García et al., 2017; Roberto & Skoglund, 1996). Some great-grandparents appreciate the limited contact due to their limited patience with young children (Doka & Mertz, 1988).

There is another reason that great-grandparenthood can be insignificant. Respondents reported that great-grandparenting could give additional meaning to their lives, but this possibility was overshadowed by other roles (e.g., parenting, grandparenting); this happened particularly in situations in which the great-grandparent had to take on the nurturing role of a parent (Barer, 2001). In most cases, great-grandparents are advanced in age and may no longer feel they are able to provide kinship care and material support; this leads them to relinquish the role of great-grandparent (Barer, 2001; Roberto & Skoglund, 1996). Most research has suggested that the great-grandparent–grandchild relationship can be characterized as similar to that of friends and mentors. For these reasons, respondents reported that the great-grandparent relationship was not fulfilled.

### Significant Role

Great-grandparenting as a meaningful and novel function has been described by Reese and Murray (1996) and Doka and Mertz (1988). Fulfilling great-grandparenthood involves an important role in maintaining family continuity and is essential for bridging the generation gap and maintaining family ties (Dolbin-MacNab et al., 2009; Lamas-Abraira, 2021). Great-grandparents can provide instrumental emotional help (Schuler, 2022) as well as financial or household help if any difficulties (e.g., health problems) arise. They can thus take full responsibility for three generations (Albrecht, 1954). According to the available research, great-grandparents are not only a source of wisdom and tradition for the family, but also an important solid structure and support. Their presence in the lives of their descendants and grandchildren has many positive aspects. Great-grandparents often pass on family rituals and traditions that are crucial for maintaining family identity and cohesion. Their advice and the wisdom they have gained from a long life can be an invaluable source of knowledge and support for younger generations in various life situations (Doka & Mertz, 1988; Dolbin-MacNab et al., 2009; [Bibr CIT0018][Bibr CIT0031]; Schuler & Brito Dias, 2023). Further, great-grandparents can provide a stable and solid family structure. Their role as authority figures and experienced mentors can help to strengthen family ties and provide a sense of security and stability for younger generations. Their presence can provide important social and emotional support for other family members (Powell, 2018; Schuler, 2022). According to Even-Zohar and (Tzurit) Garby (2016) and Doka and Mertz (1988), there are important aspects of this role:

- being a great-grandparent provided a sense of personal and family renewal and ensured the continuation of the family’s generational line.
- the role of great-grandparenthood brought welcome distractions in life, i.e., new places, new people, etc.
- becoming a great-grandparent was a sign of longevity. The satisfaction of seeing the family grow through four generations provided psychological support and created a sense of immortality that helped great-grandparents face death knowing that their family would continue (Doka & Mertz, 1988).

These studies made clear that great-grandparents have many important roles and contributions within the family. Their presence brings continuity, stability, wisdom, and support that are essential to the overall development of the family and each member (Bean et al., 2014; Brannen, 2006). The new role of a great-grandparent can provide new experiences, perspectives, and attitudes for their offspring that are different from those of their grandparents.

The main difference between the significant role of great-grandparents and their continuing role in grandparenting lies in the focus on the impact and contribution of this role. The significant role of great-grandparents emphasizes the benefits they bring to the entire family, including emotional support, the preservation of family values and traditions, and the strengthening of family stability and cohesion. This role highlights how the presence of great-grandparents enhances the quality of life for all family members, including the great-grandparents themselves. On the other hand, the continuing role in grandparenting focuses on continuity and how great-grandparents build on the role they played as grandparents. Here, the main emphasis is on preserving family values, traditions, and support across generations, ensuring that family traditions and material resources are passed down and endured. Although the significant role focuses on the active contribution to the family’s well-being and stability, the continuing role emphasizes the long-term continuity of family relationships and values.

### Common Elements for All Roles

For a better overview, the common elements for each role are shown in (correction) Table 4. The first column is a list of key themes that appear in the reviewed literature that are typical of the relationship between a great-grandchild and a great-grandparent. Each identified role (continuing, insignificant, significant) is assigned a number from 1 to 3 to indicate the importance of that characteristic:

- “**1**” = this label represents agreement with the statement listed on the left side of the table. There is a great deal of interest in instrumental help, especially among great-grandparents, whose role is new and important.
- “**2**” = this designation represents agreement with the statement, but where the great-grandparents’ options are limited primarily by health, geographic distance, or some other limitation. It is also mainly used for great-grandparents who consider their role as a continuation of grandparenthood.
- “**3**” = this label represents the lowest level of agreement with a given statement. This is especially the case with great-grandparents whose role is not considered very important.

There is not yet enough information in the available research on whether an (adult) great-grandchild has a similar normative obligation to the great-grandparents as to the grandparents. **Table 3** lists the information that can be gleaned from the literature that comprises the scoping review.

Great-grandparents play an important role in intergenerational relationships and family dynamics. They can be key actors in the lives of their great-grandchildren and provide valuable support and guidance to their offspring. To some extent, great-grandparents provide emotional and instrumental support to the family, offering advice, attention to offspring, and even financial assistance. The role of great-grandparents is in socializing, complementing grandparenting and parenting. They transmit family culture, values, and traditions. Great-grandparents are the founders of the family, providing a sense of chronology and contributing to the collective memory of the family.

## Limitations

This review has several limitations. First, there have been limited studies on determining great-grandparent roles, often with small sample sizes, making it difficult to draw definitive conclusions. The role of great-grandparents is characterized by ambiguity and few explicit expectations or prescribed behaviors (in contrast to the role of grandparents, which is usually connected with concrete normative expectations). For this reason, the role of great-grandparents in current research is typically examined in comparison to grandparenting, where roles and typologies have been adequately explored. Second, the division into three roles is limited; there is a possibility of additional roles, along with the identification of normative expectations. Future research is needed to further understand the roles and influences of great-grandparents in the lives of their great-grandchildren, especially in light of changing demographics. Third, the respondents to the studies were more often women than men. This gender imbalance may limit understanding of how care, needs, and support are negotiated in multigenerational families. Fourth, only studies that were published in English were included for review. Therefore, studies with a different language were omitted.

### Future Research

Research focused on the perspective of great-grandchildren is still limited, with most available studies primarily concentrating on the viewpoint of great-grandparents. This one-sided focus in the literature leaves room for further exploration that could delve into the perceptions and experiences of great-grandchildren, who occupy a unique position in the family hierarchy. Based on the available literature, however, it can be argued that the role of great-grandchildren is not merely passive but active, as they engage in the transmission and transformation of family values and traditions. Great-grandchildren grow up in an environment where great-grandparents often play a symbolic role as bearers of family history and representatives of the family’s longevity and stability (Doka & Mertz, 1988). Although their interactions with great-grandparents are often limited, whether due to geographical distance or the health of the great-grandparents, these encounters hold great significance for the great-grandchildren. They often value these rare moments as sources of wisdom and family identity (Barer, 2001; Roberto & Skoglund, 1996).

A specific characteristic of great-grandchildren is their ability to perceive the multigenerational perspective within the family, which allows them to better understand family dynamics and the value of intergenerational relationships. Although great-grandchildren often take on the role of listeners and receivers of stories and traditions from their great-grandparents, their own perspective remains underexplored (Drew & Silverstein, 2004; Even-Zohar & Garby, 2016). In this active role, great-grandchildren contribute to the continuity of family heritage, while also adapting and interpreting these traditions in ways that reflect their own generational identity. Exploring how great-grandchildren perceive their great-grandparents and what values and traditions they inherit from these relationships would provide important insights and fill a missing part of the overall picture of intergenerational relationships, thereby enriching the understanding of the role that great-grandchildren play within the broader family context (Knigge, 2016; Reese & Murray, 1996).

## Conclusion

The summary of studies is quite limited, but they suggest that the experiences of great-grandparents vary. Based on the literature, the scoping review distinguishes three possible roles of great-grandparenting that are influenced by several factors: related to grandparenting, insignificant, and significant. Studies show that great-grandparents engage in interactions with their great-grandchildren in a variety of activities that match those they previously shared with their grandchildren, but at a lower frequency. They pass on a legacy of values, beliefs and traditions to their offspring, thus contributing to family continuity. Great-grandparents take on different functions depending on their generational role, such as nurturing, affection, and emotional support. The role of great-grandparents is essential for maintaining family unity and conveying family history. Further research is needed to understand the details, differences, and variables that influence great-grandparenting.

## Supplementary Material

gnae181_suppl_Supplementary_Materials

## Data Availability

Supplementary data are available at The Gerontologist online.
